# A Clinical and Radiographic Evaluation of Zirconia Dental Implants: 10-Year Follow-Up

**DOI:** 10.1155/2021/7534607

**Published:** 2021-12-30

**Authors:** Andrea Enrico Borgonovo, Susanna Ferrario, Carlo Maiorana, Virna Vavassori, Rachele Censi, Dino Re

**Affiliations:** ^1^Department of Esthetic Dentistry, Istituto Stomatologico Italiano, University of Milan, Milan, Italy; ^2^Fondazione IRCCS Ca' Granda Ospedale Maggiore Policlinico, Implant Center for Edentulism and Jawbone Atrophies, Maxillofacial Surgery and Odontostomatology Unit, University of Milan, Via Della Commenda 10, Milan 20122, Italy; ^3^Head Department of Implant Center for Edentulism and Jawbone Atrophies, Maxillofacial Surgery and Odontostomatology Unit, Fondazione IRCCS Ca' Granda Ospedale Maggiore Policlinico, University of Milan, Via Della Commenda 10, Milan 20122, Italy; ^4^Head Department of Esthetic Dentistry, Istituto Stomatologico Italiano, Milan 20122, Italy

## Abstract

**Purpose:**

The aim is to evaluate the survival and success rates, as well as the marginal bone loss (MBL) and periodontal indexes, of zirconia implants with 10-year follow-up.

**Materials and Methods:**

10 patients were selected and 26 one-piece zirconia implants were used for the rehabilitation of single tooth or partially edentulous ridge. After 10 years, a clinical-radiographic evaluation was performed in order to estimate peri-implant tissue health and marginal bone loss.

**Results:**

The survival and success rates were 100%. The average marginal bone loss from baseline to 120 months after surgery was 0.92 ± 0.97 mm.

**Conclusion:**

One-piece zirconia dental implants are characterised by high biocompatibility, low plaque adhesion, and absence of microgap that can be related to the clinical success of these implants.

## 1. Introduction

To date, the use of implants for the rehabilitation of single and multiple edentulism is considered an effective and well-documented treatment.

Modern implantology has developed from the studies on bone healing and regeneration conducted in the 1950s and 1960s by the Swedish orthopedic surgeon Brånemark et al. [1]. The protocol is based on the discovery that titanium can effectively fuse with bone when osteoblasts grow on and within the raw surface of the implanted titanium. This forms a structural and functional link between living bone and the implant, defined as “osseointegration.” After establishing that the use of titanium implants is a safe rehabilitation, several modifications were made to the micro- and macrostructure of the implants in order to improve their physical, mechanical, and optical properties [2–4].

However, several disadvantages are represented by the use of a metal alloy inside the tissues. In fact, undesirable allergic reactions (0.6%) [5] have been found for which adequate epicutaneous patch tests are lacking; moreover, cell sensitization and galvanic current formation are possible [6]. In addition to these disadvantages, the possible aesthetic failure is due to the grey colouring of titanium, which is evident in thin gingival biotypes. For these reasons, the demand for more aesthetic and biocompatible implant materials has increased over time [5, 7–9].

In recent years, ceramics have occupied an important place in the dental world. In particular, the yttrium-stabilized tetragonal zirconium polycrystal (Y-TZP) has gone from being a material used solely for prosthetics to a suitable titanium substitute in implantology. There are several advantages that a material like zirconia has over titanium. First of all, there is its high biocompatibility which allows an excellent chemical stability over time and therefore solves the problem of sensitization in some patients [10, 11]. In addition, its natural white colour minimizes aesthetic failures in patients with thin gingival biotypes [12].

Secondly, in the process of osteointegration, Y-TZP allows the stimulation of osteogenic cells that combine with unique mechanical qualities such as remarkable resistance to fracture, fatigue, flexion, deterioration, and high radiopaque properties [13, 14]. In addition, zirconium oxide has been shown to moderate bacterial adhesion and excess biofilm, further reducing the likelihood of inflammation in peri-implant tissues [15–17].

On the other hand, titanium has been shown to wear when in contact with fluoride or metal alloys in saliva [18, 19]. In addition, titanium, taking on a grey colour and thus creating a dark shadow visible through the peri-implant tissues, affects the aesthetic results negatively [20–22].

On the contrary, zirconia lends itself to greater efficiency in terms of aesthetic result. Thanks to the optical behaviour of the Y-TZP, which changes according to its composition, crystal size, grain distribution, and processing methods, the material has the masking capacity, which allows dark substrates to be covered with good opacity and translucency results. This is due to the characteristics related to grain size, refractive index, absorption coefficient and residual porosity, and the existence of different additives and pigments [23–25].

The biological process of osseointegration depends on several factors which in turn are related to the implant, the position of the surgical site, the bone quality, and the surface characteristics of the implant itself [3, 26, 27]. BIC (bone-to-implant contact) is evaluated by histomorphometrically analyzing the osteointegration process at different stages of healing [27–29]. In support of this thesis, a comparable osteointegration index for zirconium implants compared to titanium implants has been demonstrated [3, 15, 28, 30–34].

However, results on implant rehabilitation with Y-TZP implants are still lacking in the long term. For this reason, the aim of the study was to evaluate the survival and success of Y-TZP implant restorations at 10 years and to analyse the stability of hard (marginal bone loss) and soft tissue over time.

## 2. Objectives

The objectives of this retrospective case series are to evaluate long-term peri-implant bone and soft tissue stability. To assess the stability of hard tissues, the distances of the bone were measured from the implant shoulder. These values are expressed with the marginal bone loss (MBL). On the other hand, to establish the stability of soft tissues, the probing pocket depth (PPD), plaque index (Pi), and probing on bleeding (BoP) were evaluated with a calibrated probe (Hu-Friedy, N. Rockwell, Chicago, IL). The data obtained were also compared to the results in the literature of titanium implants.

## 3. Materials and Methods

### 3.1. Implant System

**Figure 1:**
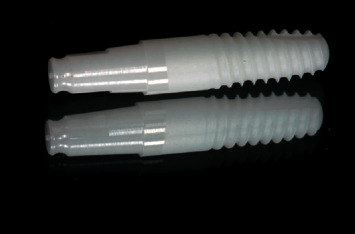
Implant characteristics.

The implants used in this study are among the first zirconia implants put on the market: they are single-component implants with conical body, double thread, and rounded apex. These are sintered and yttrium-stabilized zirconium oxide implants with an average roughness of 0.9–1 µm (Figure 1).

**Figure 2:**
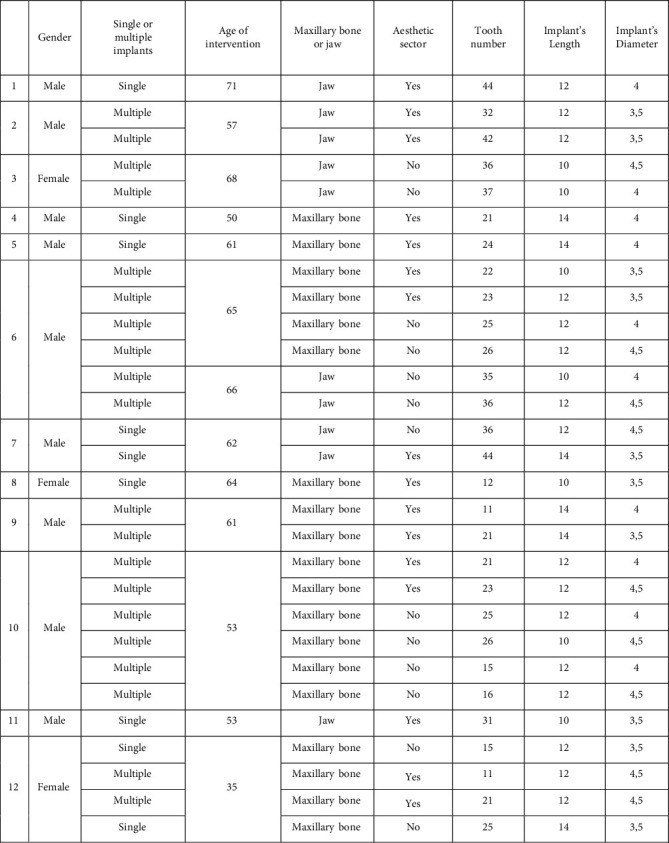
Cases selected for clinical-radiographic evaluation.

The average diameter of the implants used was 3.98 mm and the average length was 11.93 mm. 19 implants were placed in the maxilla while 10 implants were placed in the mandible (Figure 2).

### 3.2. Patient Selection

For the evaluation of radiographic bone resorption and periodontal indexes, 12 patients were selected who needed single or multiple edentulous prosthetic implant rehabilitation in the upper or lower jaw and who were rehabilitated with 29 single-component zirconia implants partially stabilized with yttrium in the period from January 2007 to April 2012. Of the 12 patients, 9 are male and 3 are female. However, for the radiographic data, there was a drop out of 2 patients for a total of 3 implants because it was not possible to find the individualized silicone bite and therefore the X-rays taken at 10 years were not standardised with those taken at the time of surgery (T0) and 1 year after surgery (T1). Therefore, the number of patients analysed with regard to MBL data is 10 for a total of 26 implants.

The inclusion criteria included the presence of a minimum bone volume of 8 mm in height and 5.5 mm in thickness at sites intended for implant treatment. Where the amount of residual bone was lower, regenerative procedures prior or simultaneous to implant insertion were associated.

Totally edentulous patients were not included in this study.

Patients younger than 18 years of age and patients with systemic disease, past or current, such as immunodeficiency, past head-neck radiotherapy, metabolic disorders, coagulopathies, treatment with bisphosphonates, cigarette smoke >10/day, and poor compliance were excluded from the protocol. Smoking is a factor that has the potential to adversely affect healing and the outcome of implant treatment [35]. In addition, the amount of daily cigarettes is directly proportional to the implant failure rate (>10 cigarettes/day). 10 years ago, these were the first clinical cases treated with monocomponent zirconia implants, and the correction of disparallelism in a full-arch rehabilitation could have created problems in the prosthetic phase. For this specific group of patients, it was preferred to avoid full-arch rehabilitation.

At the local level, all patients should not have periodontal disease in active phase and/or parafunctions, such as bruxism and sawing.

Considering the placement of the implants inside the oral cavity, 15 implants (57.7%) were placed in the aesthetic area (including all the frontal elements up to the first premolars) while 11 implants (42.3%) were placed in the posterior areas.

### 3.3. Surgical Protocol

After antibiotic prophylaxis one hour before surgery (2 g Amoxicillin with Clavulanic Acid; Augmentin, GlaxoSmithKline UK Ltd., Uxbridge), a mucoperiosteal flap is prepared. The osteotomy was then performed using rotating instruments and a surgical template to ensure that the sites were prepared correctly.

Once the site preparation was completed, the zirconium oxide implants (White-SKY, Bredent, Senden, Germany) were placed with an insertion torque of at least 35 N, verified with a torque wrench (Figure 2). In cases of multi-implant rehabilitation, the parallelism was achieved through the use of a simple surgical guide.

Through percussion, the achievement of a good primary stability was then verified. After suturing, the patient was discharged with analgesic (Paracetamol 500 mg, Angelini SpA, Ancona, Italy) and antibiotic therapy (Amoxicillin with Clavulanic Acid; Augmentin, GlaxoSmithKline UK Ltd., Uxbridge, 1 g every 12 hours for 6 days). Oral hygiene instructions were also given.

### 3.4. Prosthetic Protocol

Considering that single-component implants were used, the first postsurgical phase was the preparation of the implant abutments, with drills suitable for zirconia (Eterna, Bredent, Senden, Germany), in order to correct their inclination and height. Subsequently, the temporary restorations were relined with acrylic resin. Following occlusal controls in order to leave restorations not subjected to functional load and to avoid lateral contacts, the provisional restorations are luted with temporary cement.

For the following 6 to 8 weeks, the provisional restorations cemented on single implants were stabilized with composite wings to the adjacent teeth in order to minimize possible movement due to chewing. In fact, the presence of the abutment part penetrating into the oral cavity is a problem encountered by this type of implant, as it will be subjected to loading forces attributed to masticatory activity and tongue movements throughout the healing period [36, 37], possibly compromising the MBL.

The multiple implants were made with multiple restorations and discharged from the occlusion always to avoid occlusal overloads. The temporary prosthetic phase is always crucial also for the conditioning of the peri-implant soft tissue. After 6 months, the final crowns were made using the CAD-CAM system (LAVA, 3M ESPE, St. Paul, MN) and cemented with a glass ionomer material (GC Fuji, CEM GC America, Alsip, IL).

### 3.5. Follow-Up Protocol

Clinical-radiographic checks have been performed every 6 months since the intervention. From the periodontal point of view, the probing pocket depth (PPD), plaque index (Pi), and probing bleeding (BoP) were evaluated with a calibrated probe (Hu-Friedy, N. Rockwell, Chicago, IL).

The survival rate was indicated as the survival of the functionalized and asymptomatic implant. Alternatively, the classic criteria formulated by Albrektsson [38] regarding implant success were used.

### 3.6. Radiographic Evaluation

Standardised periapical radiographs were performed every 6 months after implant placement, using a personalised bite record made with Orthogum (Zhermack, Badia Polesine, Rovigo, Italy) and a X-ray holder.

**Figure 3:**
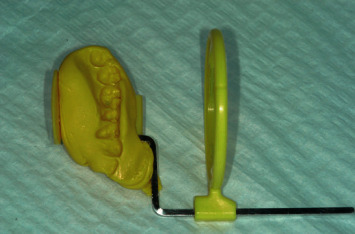
Standardised periapical radiographs were obtained using the Rinn alignment system with customised silicone bite.

**Figure 4:**
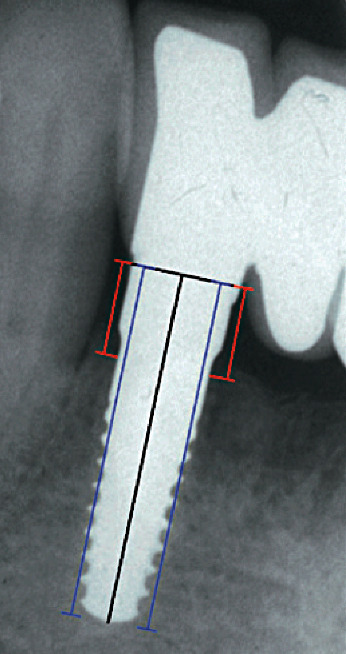
120 months after surgery: radiographic check of a zirconia dental implant positioned in 4.2. Assessment of the distance from the shoulder to the first bone-to-implant contact on digitalised radiographs (red lines). The blue lines refer to implant length.

All X-rays were subsequently converted to digital and saved in .JPG format. Analysis of the images was carried out using a software (ImageJ, US National Institutes of Health, Bethesda, Maryland, USA), thanks to which it was possible to calculate the marginal bone loss (MBL) both mesially and distally. At each revaluation, the new MBL values were compared with the baseline values (Figures 3 and 4).

### 3.7. Statistical Evaluation

Based on a descriptive statistical analysis, the mean and the standard deviation of the values obtained were calculated. The Student t-test was applied to compare the results between predefined groups (gender, age, single/multiple implants, and implant placed in the maxilla/jaw). A 95% confidence interval was applied for all measurements with statistical significance for values of *P* < 0.05. For the analyses performed on the radiographic evaluations, instead of calculating the differences, the percentages of variation of the marginal bone loss variable were calculated. In addition, the radiographic resorption values were separated into mesial and distal. The data were also finally evaluated on the total, i.e., regardless of the variables of possible confusion.

## 4. Results

With regard to the radiographic evaluation, encouraging results were found.

The average bone resorption of implants placed in the aesthetic area was 0.97 ± 1.15 mm at 10 years, considering that at 1 year it was 0.51 ± 0.91 mm, with a differential gap between 1 and 10 years of only 0.46 mm. The average bone resorption of the implants placed in posterior areas was, at 10 years, 0.72 ± 0.70 mm considering that at 1 year it was 0.53 ± 0.93 mm therefore with a differential gap between 1 and 10 years of only 0.19 mm.

In detail, it is interesting to note the difference observed for radiographic bone resorption related to single implants and multiple implants. In this study, 18 implants were connected to each other with temporary and permanent bridge restorations, while 8 implants were left individually. The multiple implants that, following placement, were connected by means of prosthetic bridges presented values of MBL, at 10 years, of 0.56 ± 0.72 mm considering that at 1 year it was 0.39 ± 0.86 mm therefore with a differential gap between 1 and 10 years of about 0.2 mm. The average bone resorption of single implants was instead, at 10 years, 1.30 ± 1.25 mm considering that at 1 year it was 0.79 ± 0.98 mm therefore with a differential gap between 1 and 10 years of about 0.51 mm.

The average bone resorption of the implants placed in the jaw at 10 years was 0.88 ± 0.72 mm considering that at 1 year it was 0.85 ± 1.10 mm therefore with a differential gap between 1 and 10 years of only 0.03 mm. The average bone resorption of the implants placed in the maxillary bone at 10 years was 0.87 ± 0.92 mm considering that at 1 year it was 0.33 ± 0.74 mm with a differential gap between 1 and 10 years of 0.54 mm.

Finally, the total average bone resorption at 10 years from time 0 was 0.92 ± 0.97 mm considering that the total average bone resorption at 1 year was 0.55 ± 0.92 mm. This means that, in general, a bone resorption of 0.37 mm was found.

However, if you want to correctly analyse the data obtained with a statistical test, the Student t-test must be applied. The confidence interval was set for all measurements to 95% and the statistically significant difference was indicated with *P*-value equal to *P*=0.05.

The analysed implants are always 26 and the peri-implant resorption levels were related to the confounding variables to observe if one of these variables had influenced the MBL in 10 years (gender, age, multiple-single implants, and implants placed in maxilla or jaw). The data were also analysed keeping the mesial and distal resorption separate. On the basis of the data obtained, it can be stated that the confounding variables do not affect the MBL variations found in the 10 years under examination.

A statistically significant difference emerges between the resorption of single implants and those with multiple anchors, with a lower resorption of the multiple anchorage implants (*P* < 0.05). However, there is no significant difference in the comparisons between implants placed in the jaw and maxillary bone and between anterior and posterior implants (*P* > 0.05).

In 6 cases, it was possible to highlight a slight bone increase that remained stable over time (≤ 0.6 mm). The positive value was recorded as early as 1 year after implant placement and remained unchanged thereafter.

**Figure 5:**
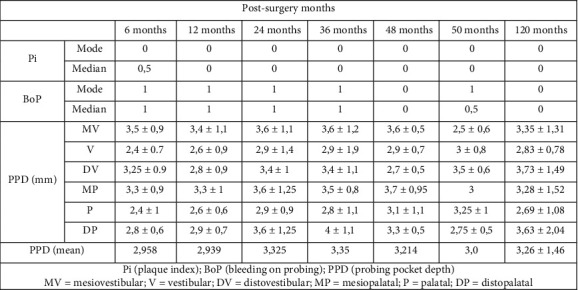
Periodontal indexes.

With regard to periodontal indices, the bleeding index (mBoP) and plaque index (mPi) peri-implant modified according to Mombelli [39], probing depth (PPD), and mobility (M) were taken into account. Evaluations were performed 6 months after surgery and then every year up to 5 years. They were then carried out again 10 years after surgery (Figure 5).

Considering an average follow-up period of 10 years, the overall bleeding rate was mBOP = 0.42 ± 0.49; considering only implants placed in the mandible, it was mBOP = 0.35 ± 0.48; regarding implants placed in the upper jaw, it was mBOP = 0.49 ± 0.50.

With respect to the peri-implant plaque index, an average value of mPi = 0.03 ± 0.17 was obtained overall. For the implants inserted in the mandible, the mean value was mPi = 0.04 ± 0.2, while in the maxillary bone the implants had a mean value of mPi = 0.03 ± 17.

Despite the presence of bleeding, only three sites were found to have a pathological PPD. No peri-implantitis was detected. The overall average depth of probing of the implants, at 120 months, was in fact equal to PPD = 3.26 ± 1.46 mm, specifically equal to 2.49 ± 1.08 mm in the mandible, and 3.66 ± 1.44 mm in the upper jaw.

## 5. Discussion

The data obtained in this study showed excellent results from the use of monocomponent zirconia implants. This preliminary clinical study presents the results of 26 dental implants placed in the anterior area of both maxillary jaws based on the concept of early loading.

From the literature analysis, there are few studies concerning the analysis of zirconia implants regarding their long-term survival and their biological and aesthetic characteristics [40, 41]. According to Oliva et al. [42], the overall success rate of 100 zirconia implants, subdivided per roughness, is 98%. Pirker et al. [43] also reported a two-year follow-up stability of the peri-implant marginal bone level in postextractive zirconia implants placed at the level of the upper first premolar.

In a recent systematic review [44], “commercially available (CA)” zirconia was considered. The study includes our own patient pool but with 4 years of follow-up [11]. From the results of the study, it can be inferred that no technical-prosthetic complications occurred. The mean bone resorption at 1 year of follow-up was 0.67 mm, which is in accordance with the literature for both zirconia implants (0.79 mm, CI 0.73–0.86) [45] and titanium implants (range 0.41–0.89 mm) [46]. In another recent systematic review [47], the marginal bone remodeling process accounted for a mean loss of 0.80 mm (95% CI: 0.60 to 1.00 mm) at a 1-year postloading period and was achieved based on the data of 10 studies. Therefore, considering the resorption observed at 10-year follow-up period of 0.92 ± 0.97 mm, it is possible to say that it agrees with the data in the literature.

Comparing the results obtained with another recent systematic review [48] of titanium implants, it can be concluded that the average MBL of titanium implants (n = 1435) was 0.98 mm at 10 years. In another prospective randomised pilot trial [49], the zirconia implants were associated with a mean MBL of 1.51 mm (SD: 0.68; median: 1.48) at 30 months and 1.38 mm (SD: 0.81; median: 1.27) at 80 months. The corresponding values for titanium implants were 0.92 mm (SD: 0.72; median: 1.03) and 1.17 mm (SD: 0.73; median: 1.05). This value is consistent with our average bone resorption data of 0.92 ± 0.97 mm.

In addition, the results of the present study have shown less bone loss around multiple zirconia implants that are connected and solidified by prosthetic bridges. Compared to single implants, multiple implants allow a more uniform force distribution. Due to the better distribution of forces, crestal bone is under less stress, resulting in better preservation of peri-implant crestal bone levels [50–52].

The promising results obtained for MBL can also be justified by the monocomponent morphology of the analysed implants. Although monocomponent implants have some limitations, they also have advantages over bicomponent implants. Indeed, the presence of a microgap at the abutment-implant interface can promote bacterial proliferation [53] as microorganisms can penetrate cracks of only 10 µm. Inflammation at the soft tissue level leads in a short time to peri-implantitis and therefore to bone loss [54, 55].

**Figure 6:**
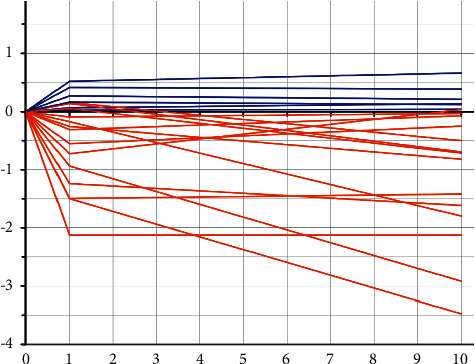
Proportion of tooth-bone distance in relation to time zero. It is possible to highlight in 6 cases a slight bone increase that has remained stable over time (<1 mm).

Radiographic evaluation showed an overall peri-implant bone loss not exceeding 1.5 mm during the first year of loading. Subsequently, from 1 year to the 10 years of follow-up, MBL values were found to meet Albrektsson's criteria [38]. To conclude, the values detected over time of peri-implant bone resorption were analysed and reported in a summary and descriptive graph (Figure 6). The study includes both anterior and posterior region. It is possible to notice how the values found at one year, in this gradual representation, show a certain trend over time. The reabsorption took place almost entirely in the first period after loading and then remained mostly stable. The 6 values can be easily seen, corresponding to 6 implants, which had a bone increase in the first year that remained stable over time.

From clinical evaluation, periodontal indexes showed an overall good level of peri-implant health. The mPI values obtained are indicative of optimal plaque control by the patients but there is a rather high level of bleeding.

The average values of PPD recorded, respectively, at 4 and 5 years of follow-up, were 3.21 and 3.0 mm and since, at 10 years, the total average was 3.26 ± 1.46, and the stability of peri-implant soft tissue to monocomponent zirconia implants is still demonstrated.

**Figure 7:**
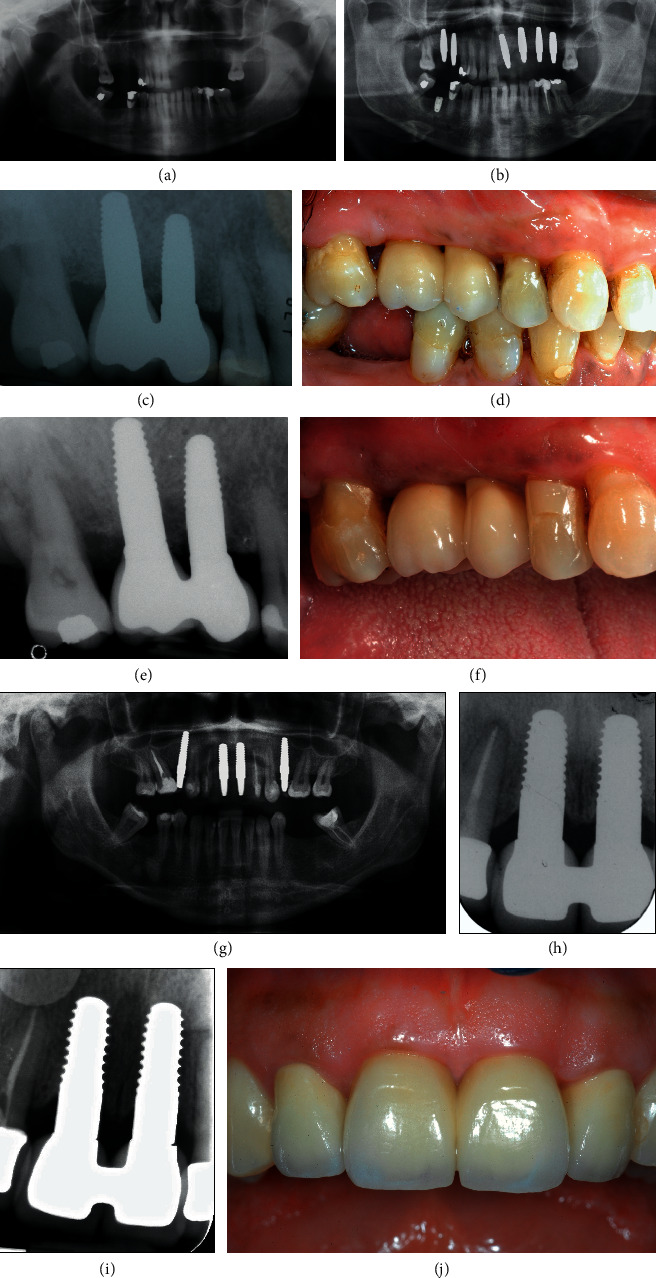
(a) Presurgery OPT. (b) Postsurgery OPT. (c) One-year follow-up RX. (d) Clinical situation at 1-year follow-up. (e) 10-year follow-up RX. (f) Clinical situation at 10-year follow-up. (g) Postsurgery radiographic situation. (h) Radiographic situation at 1 year. (i) Radiographic situation at 10 years. (j) Clinical situation at 10 years.

In addition, the literature has amply demonstrated that zirconia exhibits high biocompatibility and less plaque adhesion than titanium surfaces [56]. The long-term preservation of the peri-implant crestal bone and the health of the peri-implant soft tissue result in the maintenance of good aesthetic results over time (Figure 7).

A recent histological study reported no difference in soft tissue height between zirconium oxide and titanium implants. However, the junctional epithelium was shorter (ZrO2 = 0.76 mm vs. Ti = 1.40 mm) and had a higher collagen content in single-component zirconia implants than in single-component titanium implants [57]. To support this thesis, Cionca et al. reported a cumulative soft tissue complication rate of 0% in 76 zirconia implants placed in 52 patients [58].

Literature evaluating the soft tissue interface around implants appears to favour zirconium oxide implants over titanium, although further investigation is certainly needed [59].

In view of the results obtained from the clinical-radiographic evaluation, it was possible to define both survival and success criteria that refer to the following. The success criteria [38, 60] were formulated according to the following parameters: mobility (present not present), reported pain or paresthesia (present not present), peri-implant radiotransparency (present not present), and marginal peri-implant bone loss of less than 1.5 mm in the first year and 0.2 mm in subsequent years. Survival criteria were identified by the permanent presence of clinically asymptomatic implants under functional loading. At the end of the 10-year follow-up, 100% survival and success rates were obtained.

The objective of the present retrospective case series was to describe and report the clinical and radiographic results of zirconia dental implants. Despite this, the results presented should be considered with caution, since the included sample number was small along with the heterogeneity among the patients as far as smoking status, periodontal disease history, and medical history. Within the limitations of the present clinical evaluation in this particular population, overall, the peri-implant soft tissue and bone and prosthetic restorations were stable providing excellent clinical results and aesthetic outcomes.

## 6. Conclusion

The present retrospective case series yielded a 100% success rate for zirconia dental implants. Considering the comparable osseointegration of zirconia implants compared to titanium implants, it can be inferred from our results that zirconia implants represent a valid alternative to titanium implants, in terms of both biocompatibility and aesthetic results. The apparent reduced affinity to plaque accumulation may favour soft tissue health around zirconia dental implants and decrease the risk of inflammation or infection.

Long-term clinical studies are certainly necessary to prove our results before zirconia implants can replace titanium implants in the future. Further studies on the aging phenomenon of zirconia are also necessary to enable microstructural surface improvement of the ceramic.

However, it can be stated that zirconia implants are safe and have high long-term survival rates and low MBL values.

## Data Availability

No data were used to support this study.
